# Study and Use of Rice Husk Ash as a Source of Aluminosilicate in Refractory Coating

**DOI:** 10.3390/ma14133440

**Published:** 2021-06-22

**Authors:** Mohd Na’im Abdullah, Mazli Mustapha, Nabihah Sallih, Azlan Ahmad, Faizal Mustapha, Ayu Dahliyanti

**Affiliations:** 1 Department of Aerospace Engineering, Faculty of Engineering, Universiti Putra Malaysia, Serdang 43400, Malaysia; naimabdullah14@gmail.com (M.N.A.); faizalms@upm.edu.my (F.M.); 2 Department of Mechanical Engineering, Universiti Teknologi PETRONAS, Seri Iskandar 32610, Malaysia; nabihah.sallih@utp.edu.my (N.S.); azlan.ahmad@utp.edu.my (A.A.); 3 Department of Chemical Engineering, Universitas Pertamina, Jakarta 12220, Indonesia; ayu.dahliyanti@universitaspertamina.ac.id

**Keywords:** rice husk ash, geopolymer, response surface methodology, fire retardant, intumescent

## Abstract

The utilisation of rice husk ash (RHA) as an aluminosilicate source in fire-resistant coating could reduce environmental pollution and can turn agricultural waste into industrial wealth. The overall objective of this research is to develop a rice-husk-ash-based geopolymer binder (GB) fire-retardant additive (FR) for alkyd paint. Response surface methodology (RSM) was used to design the experiments work, on the ratio of RHA-based GB to alkyd paint. The microstructure behaviour and material characterisation of the coating samples were studied through SEM analysis. The optimal RHA-based GB FR additive was formulated at 50% wt. FR and 82.628% wt. paint. This formulation showed the result of 270 s to reach 200 °C and 276 °C temperature at equilibrium for thermal properties. Furthermore, it was observed that the increased contents of RHA showed an increment in terms of the total and open porosities and rough surfaces, in which the number of pores on the coating surface plays an important role in the formation of the intumescent char layer. By developing the optimum RHA-based GB to paint formulation, the coating may potentially improve building fire safety through passive fire protection.

## 1. Introduction

Materials’ flammability is one of the most important elements that require strict measures and precautions necessary to maintain fire safety, especially for building and construction products. This is due to the growing loss of life because of the spread of fire. Thus, comprehensive research on this issue is the main focus of this study. In order to overcome this consequence, various measuring techniques of fire properties have been developed and improved, such as lowering the heat release, controlling ignitability, or improving the extent of flame spread across the surface of flammable materials. Therefore, these factors allow a greater time for people to evacuate to safe areas before a fire takes hold and thus, saves more lives. Overall, from 2006 to 2014, the average rate of fire cases in Malaysia is up to 1024.67 fires per million people for each year [1]. From statistics, 7.53 per million populations each year is the rate of fire victims with 3.07 deaths per million populations. Hence, approximately 90 residential fires per million populations occurred each year. By taking into account fire casualties, it is about a 30% increase in the total number of victims in residential fires within 3 years [1].

In order to avoid a rise in the risk of fire, modern products, including building materials, furniture, and clothing, were mostly made of fire-retardants materials [2]. However, some of the compounds present in these materials have adverse and harmful effects on the environment, hence leading to a change to more eco-friendly alternatives in recent years [3]. Therefore, in recent years, there has been an increasing interest in the development of bio-based fire retardants. In various research, researchers have discovered that Lignocellulosic Plant Fibres (LPFs) have natural defence behaviours against the aggression of fire [4,5,6]. Rice husk (RH), which is categorised as an LPF, consists of 35% cellulose, 25% hemicellulose, 20% lignin, 3% crude protein, and 17% ash [7].

The gradual global transition from non-renewable (fossil-based) raw materials to renewable (plant-based) has intensified the search for alternative industrial raw materials. The paint industry has also not been excluded in this growing demand for renewable materials because several plant-based materials have been introduced, particularly as fillers [8]. Rice husk ash (RHA), with its known high silica content, has a vast potential for offering an alternative to commercial paint filler or additive. Currently, silica flour, kaolin, and calcium carbonate are the widely used fillers in the paint industry since these materials can be obtained naturally and have perfect crystalline silica [9]. Recent studies have shown that RHA is also suitable to be used as a filler. It is inexpensive and renewable, and most importantly, it is able to improve some mechanical properties of epoxy paints [10]. The properties of white RHA or black RHA are different because they both have a dissimilar amount of carbon and silica due to different pre-treatments of rice [10]. Industrials have assured the potentials of rice husk (RH) on various applications due to its high silica content. The applicable way to use RHA as an extender along with paint has been investigated in some paints, namely, textured emulsion, cellulose matt paint, and matt wood varnish [11]. However, there is still not as yet well-established research in adding RHA-based geopolymer into alkyd paint, proven by application.

Geopolymer is known for its excellent properties; thus, it has been applied in several industrial applications and has attracted global investments with commercialisation in many categories, namely, resin, paint, binder, grout, cement, concrete, ceramic, panels, and fibre-reinforced composites [12]. Geopolymer binder (GB) has been verified to demonstrate outstanding fire resistance properties [13], high mechanical strength [14], high durability [15], and numerous aluminosilicates have been used, such as fly ash, metakaolin, palm oil fuel ash (POFA), and dolomite [16]. However, the GB used can cause an increase in substrate weight, darker colour, and uneven surface. These effects should be considered and not be ignored because they give rise to other issues after the application. Thus, the utilisation of RHA-based GB in coating application is low. The effective use of RHA-based GB in paint, therefore, needs to be studied so that an efficient coating produced can have a maximum advantage. 

Accordingly, it is valuable to discover cheap and renewable resources to be applied to the construction sector. RHA, which is a leftover outcome from rice granulating, has a high potential to be the alternate resource for silica [10]. Therefore, it is important to utilise RHA since it is an abundant source, in Malaysia particularly. In Malaysia, the Food and Agriculture Organization (FAO) reported that rice-paddy production is estimated to increase by 0.1 tonnes every year [17]. The growing demand for rice paddy produced about 0.52 tons of RH annually [18], with about 20% of the total grain weight obtained from the average husk weight. Therefore, approximately 200 kg of husk is produced from a tonne of rough rice, which is considered as the biodegradable waste product in the rice mill industry and is commonly burned in the open area or dumped in landfills [18].

Due to potential explored in previous studies, RHA could be the next generation of geopolymer technology and environmentally friendly sources for paint filler or additive. In this study, a new-fangled coating with RHA-based GB was developed particularly for fire-resistant steel application. This coating is expected to contribute to high mechanical properties and auspicious fire-resistance properties. Thus, an eco-friendly, efficient, and green product can be produced and has extensive potential application in the industry. The objective of this research is to optimise the RHA-based geopolymer ratio using response surface methodology (RSM) and study the effect of RHA-based geopolymer addition on alkyd paint coating in terms of thermal properties.

## 2. Materials and Methods

### 2.1. Design of Experiment Based on RSM

Two factors are chosen; namely, the RHA-based geopolymer, which acts as a fire retardant (FR), and paint, designated as A and B, respectively. The desired responses were the time taken to reach 200 °C (TT200) and temperature at equilibrium (TAE) that were assumed to be influenced by the two factors. Levels of the three chosen factors and their working ranges are shown in Table 1. The RSM of Design Expert software version 11 (Stat-Ease Inc., Minneapolis, MN, USA) was used in this study.

The central composite design (CCD) was chosen over the Box–Behnken design since it provided better information on the function within the region of experimentation and was able to make a better prediction, as reported in previous literature [19]. The significance of the different models (linear, two-factorial, quadratic, and cubic) was determined by using both F-test and *p*-value. Results showed that the quadratic model most suitably described the relationship between variables and responses. The experimental data of the historical design experiment is represented in the general form of the quadratic model. The validity of the quadratic model was expressed by the coefficient of determination, R2, and the coefficient of adjusted determination, Adj-R^2^, while statistical significance was verified with the F-test and the adequate precision ratio.

### 2.2. Raw Materials 

Rice husk (RH) in this research was obtained from a local rice factory, which is located at Tanjung Karang, Selangor, Malaysia. The species of the RH obtained was Oryza sative (Asian rice), which is vastly cultivated all over Asia. The RH was vetted to remove contaminations such as sands, rocks, and rice straws. In this process, the RH was washed and soaked in distilled water for 2 h in a large container to remove dirt and possible contaminations. Clean RH can be obtained as it floats on the surface of the water and was transferred to a sieve for the drying process and was left to dry for 24 h at room temperature. The next day, the RH samples were further dried in an oven at 100 °C for 24 h to ensure fully dried RH was obtained. For the incineration process, WiseTherm Digital Muffle Furnace (Daihan, Gangwon, Korea) was used to produce RHA with a controlled temperature of 600 °C for two hours. RHA was first ground using pulverising machine RT-02A (Mill Powder Tech, Tainan, Taiwan) and sieved using Endecotts Laboratory Test Sieve (Endecotts, London, UK) to obtain an average particle size below 65 μm. From the particle size distribution, the sample was polydispersed and had a size distribution at 0.067 μm to 56.63 μm. The chemical compositions of RHA are shown in Table 2.

### 2.3. Preparation of Geopolymer Binder Hybrid Paint Coating

**Figure 1:**
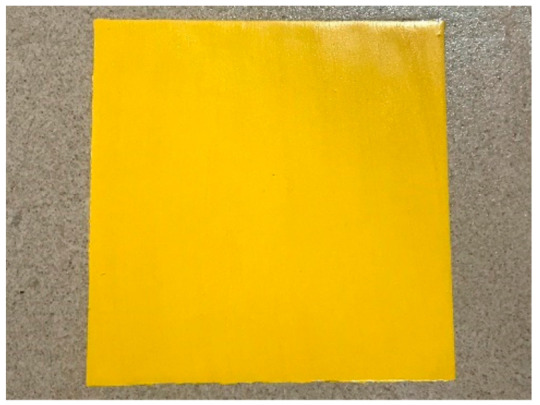
Geopolymer binder hybrid paint coating sample.

In order to form the geopolymer binder, the optimum ratio of NaOH and NA_2_SiO_3_, which acts as the Activated Alkaline (AA) solution, was mixed with RHA. Na_2_SiO_3_ solution was purchased from LGC Scientific. NaOH pellets with 97% purity were provided by Merck KGaA (Darmstadt, Germany). Concentrations, which are expressed as molarity, 8M of NaOH solution, were prepared based on the number of pellets dissolved in de-ionised water. These solutions were mixed based on the previous study [13]. The produced RH-based geopolymer binder is denoted as FR in this study. In this research, Na_2_SiO_3_ was added into the NaOH solution at a ratio of 5.5 to form an optimal AA solution [13], while the optimal ratio of AA to RHA is 2.5 to form an optimal FR additive. A total of 39 pieces of mild steel plates were cut into 100 mm × 100 mm × (1.0 ± 0.3 mm) and were cleaned with acetone to ensure the plates were free from grease or oil. A yellow (BS2660-0001 Canary) Super Gloss Finish 6000-S Alkyd based paint was used throughout this experiment. The primary reason this colour was chosen is due to most steel and machinery being coated in yellow colour for caution and visibility. A sample of geopolymer binder hybrid paint is shown in Figure 1.

### 2.4. Microstructure Analysis and Material Characterisation

SEM was used to analyse external morphology such as texture, chemical composition, crystalline structure, and the orientation of the materials of the samples. The microstructure was outlined by exposing the surface structure of material underneath a microscope with at least 25× magnification. SEM imaging was operated using a Hitachi S-3400 N (Hitachi, Tokyo, Japan) for the particle study and compound representation of the samples. It was conducted at 15 kV. The importance of the SEM result is used to prove and support the information about the phase or structure of RHA conducted in the X-ray Diffraction test. Thermogravimetry analysis (TGA) was carried out using Mettler Toledo micro and ultra-micro balances in an atmosphere of flowing nitrogen gas in alumina crucibles at a heating rate of 10 °C/min over a temperature range from 50 °C to 1000 °C.

### 2.5. Fire Resistance Test

**Figure 2:**
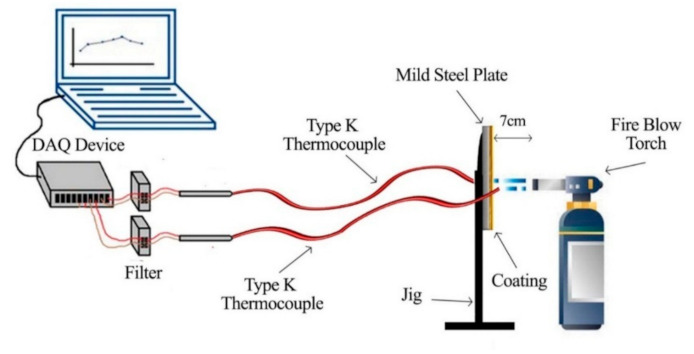
The fire resistance test illustration.

The fire resistance test was performed until the backside of the paint coating reached failure temperature. The fire resistance testing was designated using a thermocouple that acted as a temperature sensor to check the temperature at the backside of the coating samples. The test was carried out by using two Type-K thermocouples connected to a DAQ sensor. The thermocouples were attached at the backside of the steel plate coated with a fire-retardant coating (to measure the heat on the backside) and at the front of the surface of the coating (to measure the initial temperature produced by the fire blowtorch). The coatings were exposed to heat with a temperature from 800 to 1000 °C for 60 min, which complies with BS 9999: Code of practice for fire safety in the design, management, and use of buildings (BSI, 2008). The test produced time/temperature graphs and the visual effects for all samples. An illustration of the fire resistance test is shown in Figure 2. The temperature at equilibrium (TAE) and time taken to reach 200 °C (TT200) were the results obtained from the time–temperature curve.

## 3. Results

The complete design matrix and responses values of the time taken to reach 200 °C (TT200) and temperature at equilibrium (TAE) are given in Table 3.

### 3.1. Statistical Analysis of Thermal Properties

In the Design-Expert software, the fit summary tab proposes the highest order polynomial, where the additional terms are significant, and the model is not aliased. The sequential F-test for the significance of both the regression model and the individual models’ terms, along with the lack of fit test, were carried out. The ANOVA analysis for TT200 for the quadratic model in Table 4 summarises the response analysis and the significant model term. *p*-values less than 0.0500 indicate that model terms are significant, which in this case, A, B, AB, A^2^, and B^2^ are significant model terms. The predicted R^2^ of 0.9012 is in reasonable agreement with the adjusted R^2^ of 0.9251, as the difference is less than 0.2. This indicated that 90.12% of the sample variation in the response was attributed to the factors. A ratio of 31.898, which is greater than 4, indicates logical agreement and significant relationships.

Meanwhile, ANOVA analysis for TAE in Table 5 shows that all factors and interaction effects were significant, with *p* < 0.0500 except for AB with a *p* value of 0.8052 and B^2^ with a *p* value of 0.0559. If there are many insignificant model terms, a model reduction may improve the model. The predicted R^2^ of 0.9346 is in reasonable agreement with the adjusted R^2^ of 0.9517, where the difference is less than 0.2. This indicated that 93.46% of the sample variation in the response was attributed to the factors. A ratio of 42.244, which is greater than 4, indicates logical agreement and significant relationships.

The regression models can be used to calculate and analyse the effect of factors on the fire resistance performance of paint mixed with the FR additive. The equations are presented in terms of coded factors and actual factors, which are useful to make predictions about the response for the given levels of each factor.

Regression models for TT200 and TAE are respectively expressed in terms of actual factors.

Y_TT200_ = 340.46 + 2.86 (A) − 5.37 (B) − 0.07 (AB) + 0.09 (A^2^) + 0.04 (B^2^)

Y_TAE_ = 607.43 − 11.91 (A) − 2.80 (B) + 0.01 (AB) + 0.07 (A^2^) + 0.04 (B^2^)

This regression model can be used to calculate and analyse the effect of factors on the thermal properties of RHA-based geopolymer hybrid paint. 

### 3.2. Effect of Factors on Thermal Properties

**Figure 3:**
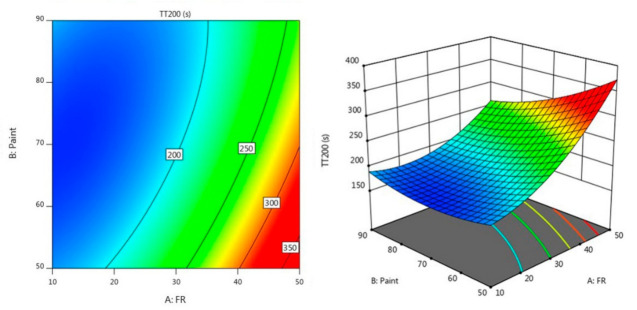
Contour plot for the effect of FR and paint on TT200 in the fire resistance test.

**Figure 4:**
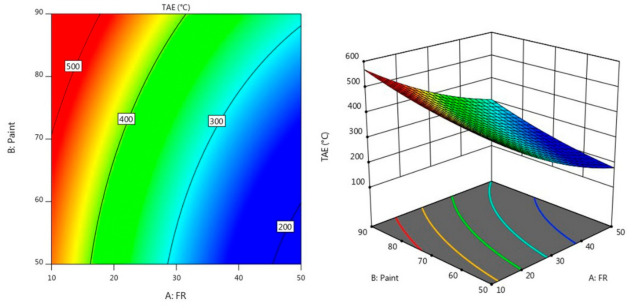
Contour plot for the effect of FR and paint on TAE in the fire resistance test.

Figure 3 and Figure 4 describe the contour diagram for the response model for the time taken to reach 200 °C (TT200) and temperature at equilibrium (TAE), respectively. As indicated by the colour key, it shows that a higher percentage of A and a lower percentage of B could result in a longer TT200, which is above 350 s. Meanwhile, a higher percentage of A and a lower percentage of B resulted in a lower temperature at equilibrium, which is below 200 °C.

### 3.3. Optimisation of the Responses for Thermal Properties

**Figure 5:**
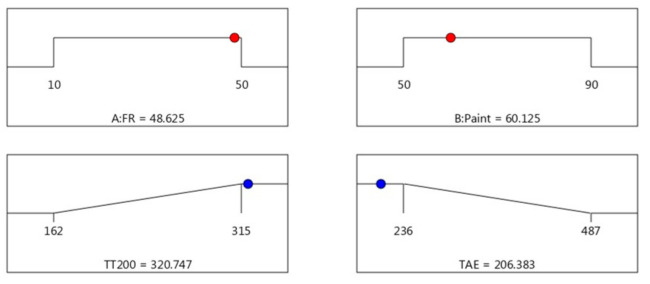
Optimum conditions and response for thermal properties.

Since the objective was to maximise the time taken to reach 200 °C and minimise the temperature at equilibrium (TAE), the maximum acceptable value for TT200 was set at 315 s, and the minimum acceptable value was set to 162 s. Meanwhile, for TAE, the target values, which is the minimum temperature at equilibrium, were set at 236 °C, and the maximum acceptable value was set for 487 °C. Figure 5 illustrates the predicted optimum conditions and the responses studied for thermal properties. The predicted optimum operating parameters influencing thermal properties was estimated to be FR (48.625% wt.) and paint (60.125% wt.). At these optimum conditions, the corresponding predicted TT200 and TAE was found to be 320 s and 206 °C, respectively. The desirability of optimisation was calculated as 1.000, indicating that all parameters were within the target to obtain the maximum fire resistance properties.

### 3.4. Experimental Validation

Experimental validation is the final step in the modelling process, and it verifies the model’s accuracy. Three validation experiments were carried out under the optimal conditions obtained from the optimisation plot, as shown in Figure 3, in order to verify the reproducibility of the established regression model and the RSM model. For a nonlinear process, the optimisation and validity of the model are only verified when the average difference between experimental and predicted values is less than 15% [19]. Table 6 shows the experimental validation for fire resistance properties; it was found that the average errors for the TAE and TT200 were well below 15% at 4.58% and 6.47%, respectively. It was concluded that the developed regression model established using this method was able to optimise the value for the responses.

### 3.5. Fire Resistance Performance

**Figure 6:**
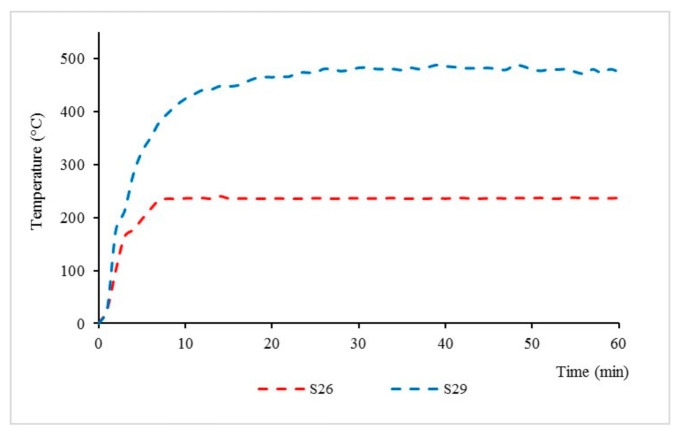
S26 and S29 fire resistance test performance.

Design matrix and responses values for sample S26 and S29 were discussed in detail and are shown in Figure 6; these samples exhibited the best and worst performance in thermal properties, respectively.

For TAE response, sample S26 showed a 103.81% improvement when compared to sample S29. Meanwhile, for the TT200 response, sample S26 showed a 94.44% improvement when compared to sample S29. From this test, it can be seen that the equilibrium temperatures and time taken to reach 200 °C for sample S26 were significantly lower compared to sample S29 due to the positive synergistic effect of the additive on fire resistance [20].

From the fire resistance, test it is noted that intumescent starts to occur at the time of 10 min for sample S26. Once the coated surface was exposed to fire, it started to melt and become a highly viscous liquid. Chemical reactions took place, leading to bubble formation, which then produced swelling and a porous intumescent char layer. A swelling char layer was formed, which minimised the heat transfer from the heat source to the underlying steel and maintained the integrity of the protected substrate against fire. 

As for sample S29, there is no formation of an intumescent char layer observed. This is mainly due to the ratio of FR to paint in this sample being very minimal, 10:70, which causes a longer decomposition time and affects intumescent formation. Regardless of its unformed intumescent char formation, chemical reactions in the paint coating led to bubble formation, which helps to minimise the smoke emission and prevent fire ignition during the fire resistance test. This is mainly due to RHA physical properties, which contain a high amount of silica. According to Sekifuji et al. (2017), silica in RH is a useful material, which offers flame resistance and antioxidation properties in coatings [21]. 

**Figure 7:**
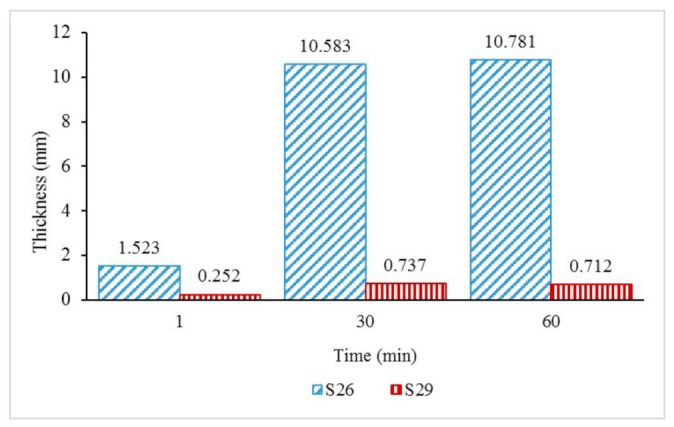
S26 and S29 char layer thickness with increasing time.

The intumescent char layer thickness of samples S26 and S29 was further investigated in Figure 7 to study the effect of fire on the intumescent char thickness. The thickness of the coatings was measured at the start of the test (1 min), during the test (30 min), and after the fire resistance test (60 min). From observations in Figure 5, sample S26 started to swell rapidly once exposed to fire and formed a 1.523 mm-thick char layer after 1 minute. As the time and temperature increased, sample S26 continued to swell and formed thicker char layers of 10.583 mm and 10.781 mm, respectively, at 30 and 60 min. Meanwhile, for sample S29, the coating only formed 0.252 mm of char thickness after a minute. It was observed that the char thickness during the test at 30 min (0.737 mm) decreased at the end of the test (0.712 mm). This indicated that the coating was unable to withstand the increasing temperature and experienced a substantial mass transfer out from the char and increased the heat transfer into the metal surface.

The results indicated that the 50.0% weight of the FR addition contributed significantly to better fire protection efficiency due to the formation of the char layer, which affects the equilibrium temperature. The result is in agreement with Beh et al. (2019) in their study [22], which shows that the char layer’s thickness influenced the coating’s fire-safety efficiency, and there was a correlation between the char layer’s thickness and the equilibrium temperature.

### 3.6. Material Characterisation and Microstructural Analysis

**Figure 8:**
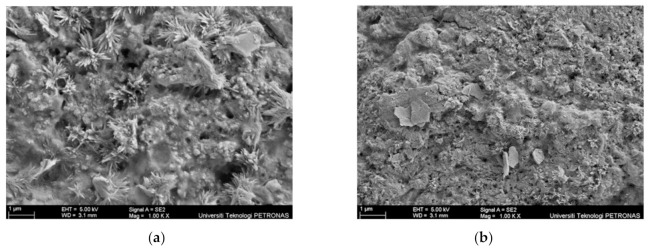
Coating surface sample before the fire test: (**a**) S26 and (**b**) S29.

Two samples; specifically, sample S26, which produced good fire resistance performance, and S29, with poor fire resistance performance, were selected for further characterisation and microstructural analysis. The surface of the coating samples for sample S26 and S29 before the fire test was analysed using an SEM micrograph, as shown in Figure 8.

The main difference between samples S26 and S29 can be seen clearly based on the unreacted particles’ amount and the surface roughness on the coating surface before the fire test. In Figure 8a, sample S26 showed a rough and porous surface due to the RHA amount, while in Figure 8b, sample S29 showed a greater amount of unreacted particles, which indicated that the combination of RHA and AA fillers did not fully dissolve in alkyd paint, hence resulting in an uneven distribution and rougher surface finish. This evidence was supported by the EDX test result in Table 7. Samples for this test were taken from the upper surface of the coating, which was directly exposed to the fire. The difference in wt. % between Si and Na content in sample S29 was higher, 7.01%, compared to sample S26 (3.69%), indicating that the RHA particles may not have fully dissolved, resulting in an uneven distribution of the additive, which caused the formation of a rough surface on the coating.

**Figure 9:**
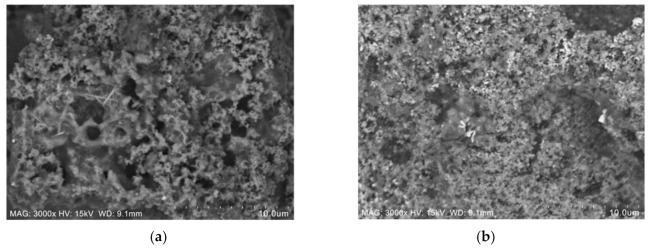
Coating surface sample after the fire resistance test: (**a**) S26 and (**b**) S29.

Sample S26 and S29 after fire resistance test were then further analysed using SEM micrographs, as shown in Figure 9. 

Samples for these tests were taken from the upper surface of the coating, which was directly exposed to the fire. From the SEM observation in Figure 9a, sample S26 contains long-rod structures, which were a certain form of sodium derived from Na_2_SiO_3_ or due to a side reaction of NaOH and RHA [13]. The number of pores on the coating surface plays an important role in the formation of an intumescent char layer. Evaporated water molecules are alleged to travel from the exposed surface of the hot fire to the cooler inner part of the material. The pressure in the pores of the material and in the microvoids was generated by water due to the rapid evaporation rate [23]. Since crystallisation of the surface and the intumescent process successfully took place in the sample, the evaporated water molecules were transported to a cooler area of the material, resulting in very low temperature at the non-exposed area. Once the coating was exposed to heat, water filled the pores to form an intumescent layer. This can be seen in the surface of the coating after the fire test, once exposed to heat, where sample S26 started to swell and formed a char layer, protecting the mild steel substrates. Meanwhile, sample S29 in Figure 9b showed a small number of pores comparatively and failed to form an intumescent char layer due to a minimal amount of FR additive. Elements’ presence in sample S26 and S29 after the fire test were then investigated using EDX and tabulated in Table 8. The major elements present in the coating were carbon (C), oxygen (O), sodium (Na), and silica (Si).

From the EDX results in Table 8, sample S26 contained 47.00% of the element of oxygen (O), carbon (C) 28.59%, silica (Si) 18.72%, and sodium (Na) 5.69%. According to Zhao et al. (2009), Si content in RHA plays a major role in the fire-resistance effect. Si contributes to the formation of a silica–ash layer which acts as a heat barrier. This layer is important in restricting the access of oxygen to the inner part of the coating, which helps to slow down the gasification process. The sufficient loading, uniform dispersion, and integrity of the silica ash layer influence the effectiveness of the fire resistance properties. Thus, this explains the high element of oxygen presence in sample S26 after the fire test. The presence of sodium (Na) also can be confirmed by the presence of the long-rod structure in the Figure 9a SEM image. As reported by previous studies [13,24,25], the long-rod structures were a certain form of sodium derived from Na_2_SiO_3_ or due to a side reaction of NaOH and RHA.

Meanwhile, sample S29 showed 45.56% of the element of oxygen (O), followed by carbon (C) 25.80%, sodium (Na) 11.22%, and silica (Si) 17.42%. Better fire-protection performance can also be determined by the antioxidant properties of the intumescent coating. Better antioxidant properties are demonstrated by coatings that have lower oxygen to carbon ratios. By comparing oxygen to carbon ratios for both samples, sample S26 has a better antioxidant properties value of 1.643, compared to sample S29 with 1.766. Thus, results from EDX analysis support the results obtained from the fire resistance test, as sample S26 showed better fire protection performance and is matched with the result obtained from EDX analysis.

### 3.7. Thermogravimetric Analysis (TGA)

**Figure 10:**
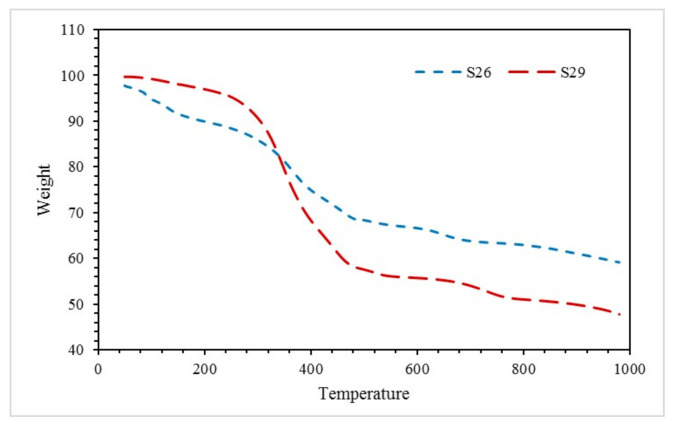
TGA of sample S26 and S29 in the fire resistance test.

Thermal degradation of sample S26 and S29 was analysed using the TGA test. Figure 10 shows TGA results obtained when samples were exposed to nitrogen at a heating rate of 10 °C/min over a temperature range from 50 °C to 1000 °C.

At the beginning of the experiment (50–200 °C), sample S26 recorded a rapid weight loss of 7.856%, which can be linked with the removal of free water, bonded water with hydrogen bond [26], and water bonded to silicate molecules [27]. Water loss through endothermic dehydration left behind a thermally stable residue [28]. The small weight loss at this stage can also be related to the softening stage of solvent vaporisation [29]. Meanwhile, for sample S29, weight loss occurred at 50–200 °C was 2.704%. Minimal weight loss for sample S29 can be associated with the RHA-based GB ratio, as this sample contained only 10 wt. % FR and 70 wt. % paint. As the endothermic dehydration reaction took place early, this result was reflected in the fire resistance test, which revealed that sample S26 intumesced faster, thicker, and possessed good fire-resistant properties.

The subsequent stage of the TGA curve (200–500 °C) is the stage of oxidative degradation for alkyd paint [30]. It was observed that sample S26 recorded a 21.56% weight loss. Meanwhile, sample S29 recorded a 39.39% weight loss. Although sample S29 recorded a large weight loss at this stage, this sample exhibited poor thermal properties since the intumescent process was not fully able to take place due to minimal water content evaporated when exposed to the fire and generated less pressure in the pores of the material for thermal expansion.

The final stage of the TGA curve (500–1000 °C) displayed the region where the material approached thermal stability. By investigating the rate of water loss between 500 and 1000 °C for each sample, sample S26 indicated a lower rate of water loss with 0.009216 (%/°C) compared to sample S29 with 0.00976 (%/°C). This shows that sample S26 might reach thermal stability faster at a temperature over 1000 °C compared to sample S29.

Furthermore, at the end of the experiment, the residual weight of the coating for samples S26 and S29 was 59.15% and 47.81%, respectively. This indicated that sample S26 decomposed minimally compared to sample S29. This phenomenon was due to the catalyst effects of the FR additive. The presence of the RHA-based GB has led to improved thermal stability and fire retardancy of the coating sample. Furthermore, a suitable amount of RHA-based GB can enhance the thermal stability of the alkyd paint, which in this case was 50 wt. % FR and 70 wt. % paint.

By taking into account the thermal degradation and thermal stability of each sample, sample S26 possessed the best thermal performance as the sample that successfully thermally degraded and formed the intumescent layer during the fire resistance test, which helps to protect the mild steel substrate and lowered the heat of the sample at a much faster rate of time compared to other samples.

## 4. Conclusions

The experimental works using RSM have successfully identified the significant factors and optimised the responses. Experiments on the thermal properties conducted were based on the design matrix generated by the Design Expert version 11 software (Stat-Ease Inc., MN, USA) and then carried out on the laboratory scale following the appropriate standards. Based on the thermal test, the outcomes demonstrated that the coating sample contained 50.0% weight FR and 70.0% weight paint significantly contributed to a better fire protection performance due to the formation of intumescent, which helps to protect the mild steel substrate, thus affecting the temperature at equilibrium and time taken to reach 200 °C. For both TAE and TT200 responses, sample S26 showed 103.81% and 94.44% improvement when compared to sample S29. Furthermore, the increased contents of RHA showed an increment in terms of the total and open porosities and rough surfaces, in which the amount of pores on the coating surface plays an important role in the formation of the intumescent char layer. In the TGA test, sample S26 possessed the best thermal performance, as the sample reached thermal stability at a faster rate and decomposed minimally compared to other samples. This phenomenon was due to the catalyst effects of the FR additive. The presence of the RHA-based GB addition has led to improved thermal stability and fire retardancy of the coating sample.

## Figures and Tables

**Table 1:** Factors and levels used for the fire resistance test.

Factor	Symbol	Levels				
		−1	−0.5	0	0.5	1
FR	A	10	20	30	40	50
Paint	B	50	60	70	80	90

**Table 2:** Chemical composition of RHA.

Element	SiO_2_	K_2_O	Al_2_O_3_	Fe_2_O_3_	CaO	Others	LOI
(wt.%)	90.58	0.82	0.06	0.03	0.02	0.02	8.47

LOI = Loss ignition, Others = Element containing TiO_2_, MgO, Na_2_O, and MnO.

**Table 3:** Design matrix and response value for the fire resistance test.

Sample	Coded Factors		Uncoded Factors		Responses		Sample	Coded Factors		Uncoded Factors		Responses	
	A	B	A	B	TT200	TAE		A	B	A	B	TT200	TAE
S1	−0.500	0.500	20	80	168	439	S21	0.000	0.000	30	70	202	332
S2	0.000	−1.000	30	50	246	274	S22	0.000	0.000	30	70	201	331
S3	−0.500	−0.500	20	60	192	421	S23	0.000	0.000	30	70	198	330
S4	0.000	1.000	30	90	197	425	S24	−1.000	0.000	10	70	164	481
S5	0.500	0.500	40	80	198	285	S25	−0.500	0.500	20	80	172	445
S6	0.500	0.500	40	80	201	287	S26	1.000	0.000	50	70	315	236
S7	0.000	0.000	30	70	198	330	S27	1.000	0.000	50	70	314	237
S8	0.000	0.000	30	70	198	333	S28	0.500	−0.500	40	60	251	261
S9	0.000	0.000	30	70	201	331	S29	−1.000	0.000	10	70	162	487
S10	0.000	0.000	30	70	209	337	S30	0.000	0.000	30	70	199	331
S11	0.000	0.000	30	70	199	331	S31	0.000	0.000	30	70	202	336
S12	0.000	0.000	30	70	201	334	S32	0.000	0.000	30	70	200	330
S13	0.500	−0.500	40	60	246	258	S33	0.500	−0.500	40	60	248	259
S14	0.000	0.000	30	70	199	330	S34	−0.500	0.500	20	80	173	441
S15	0.000	0.000	30	70	201	333	S35	0.000	0.000	30	70	202	332
S16	−0.500	−0.500	20	60	190	419	S36	0.000	1.000	30	90	195	425
S17	0.000	1.000	30	90	195	423	S37	0.000	−1.000	30	50	243	269
S18	1.000	0.000	50	70	312	239	S38	0.500	0.500	40	80	203	287
S19	0.000	−1.000	30	50	240	266	S39	−0.500	0.500	20	80	163	483
S20	−0.500	−0.500	20	60	186	418							

TAE = Temperature at equilibrium (in °C); TT200 = Time taken to reach 200 °C (in seconds).

**Table 4:** ANOVA analysis for TT200.

Source	Sum of Squares	Degree of Freedom	Mean Square	F-Value	*p*-Value	
Model	51,198.27	5	10,239.65	94.84	<0.0001	Significant
A	38,025.00	1	38,025.00	352.20	<0.0001	
B	6453.44	1	6453.44	59.77	<0.0001	
AB	645.33	1	645.33	5.98	0.0200	
A^2^	5821.49	1	5821.49	53.92	<0.0001	
B^2^	1362.82	1	1362.82	12.62	0.0012	
Residual	3562.81	33	107.96			
Cor Total	54,761.08	38				

**Table 5:** ANOVA analysis for TAE.

Source	Sum of Squares	Degree of Freedom	Mean Square	F-value	*p*-Value	
Model	199,263.77	5	39,852.75	150.83	<0.0001	Significant
A	163,216	1	131,500	497.55	<0.0001	
B	32,160.44	1	17,667.06	66.87	<0.0001	
AB	16.33	1	16.33	0.0618	0.8052	
A^2^	3622.50	1	3622.50	13.71	0.0008	
B^2^	1037.17	1	1037.17	3.93	0.0559	
Residual	8719.15	33	264.22			
Cor Total	207,982.92	38				

**Table 6:** Experimental validation for the fire resistance test.

	TT200 (s)			TAE (°C)		
	Experimental Value	Predicted Value	Error (%)	Experimental Value	Predicted Value	Error (%)
SV_1_	298	320	6.88	227	206	10.19
SV_2_	311	320	2.81	212	206	2.91
SV_3_	307	320	4.06	219	206	6.31
	Error		4.58	Error		6.47

SV = Sample validation.

**Table 7:** EDX results before the fire resistance test.

Element	S26	S29
	wt. %	wt. %
C	12.92	26.09
O	52.53	52.34
Na	15.43	7.28
Si	19.12	14.29

**Table 8:** EDX results after the fire resistance test.

Element	S26	S29
	wt. %	wt. %
C	28.59	25.80
O	47.00	45.56
Na	5.69	11.22
Si	18.72	17.42

## Data Availability

All data are presented in the article.
